# The Expression of Connexin 37, 40, 43, 45 and Pannexin 1 in the Early Human Retina and Choroid Development and Tumorigenesis

**DOI:** 10.3390/ijms23115918

**Published:** 2022-05-25

**Authors:** Matea Žužul, Mirela Lozić, Natalija Filipović, Samir Čanović, Ana Didović Pavičić, Joško Petričević, Nenad Kunac, Violeta Šoljić, Mirna Saraga-Babić, Suzana Konjevoda, Katarina Vukojevic

**Affiliations:** 1 Department of Ophthalmology, General Hospital Zadar, 21000 Split, Croatia; matea90zuzul@gmail.com (M.Ž.); canovicsamir@yahoo.com (S.Č.); adidovic@yahoo.com (A.D.P.); suzana.konjevoda@gmail.com (S.K.); 2 Department of Anatomy, Histology and Embryology, School of Medicine, University of Split, Šoltanska 2, 21000 Split, Croatia; mirela.lozic@mefst.hr (M.L.); natalija.filipovic@mefst.hr (N.F.); msb@mefst.hr (M.S.-B.); 3 Department of Health Studies, University of Zadar, 23000 Zadar, Croatia; 4 Department of Pathology, School of Medicine, University of Mostar, 88000 Mostar, Bosnia and Herzegovina; josko.petricevic@mef.sum.ba; 5 Department of Pathology, University Hospital of Split, 21000 Split, Croatia; nenad.kunac@mefst.hr; 6 Department of Histology and Embryology, School of Medicine, University of Mostar, 88000 Mostar, Bosnia and Herzegovina; violeta.soljic@mef.sum.ba; 7 Faculty of Health Studies, University of Mostar, 88000 Mostar, Bosnia and Herzegovina; 8 Department of Anatomy, School of Medicine, University of Mostar, 88000 Mostar, Bosnia and Herzegovina

**Keywords:** eye development, retinoblastoma, choroid melanoma, connexin 37, connexin 40, connexin 43, connexin 45 and pannexin 1

## Abstract

The expression pattern of Connexins (Cx) 37, 40, 43, 45 and Pannexin 1 (Pnx1) was analyzed immunohistochemically, as well as semi-quantitatively and quantitatively in histological sections of developing 8th- to 12th-week human eyes and postnatal healthy eye, in retinoblastoma and different uveal melanomas. Expressions of both Cx37 and Cx43 increased during development but diminished in the postnatal period, being higher in the retina than in the choroid. Cx37 was highly expressed in the choroid of retinoblastoma, and Cx43 in epitheloid melanoma, while they were both increasingly expressed in mixoid melanoma. In contrast, mild retinal Cx40 expression during development increased to strong in postnatal period, while it was significantly higher in the choroid of mixoid melanoma. Cx45 showed significantly higher expression in the developing retina compared to other samples, while it became low postnatally and in all types of melanoma. Pnx1 was increasingly expressed in developing choroid but became lower in the postnatal eye. It was strongly expressed in epithelial and spindle melanoma, and particularly in retinoblastoma. Our results indicate importance of Cx37 and Cx40 expression in normal and pathological vascularization, and Cx43 expression in inflammatory response. Whereas Cx45 is involved in early stages of eye development, Pnx1might influence cell metabolism. Additionally, Cx43 might be a potential biomarker of tumor prognosis.

## 1. Introduction

The vertebrate retina is a multi-layered structure with a large diversity of component cells that form morphologically and functionally distinct circuits that work in parallel, and in combination, to produce a complex visual output [1]. Adjacent to the retina is the middle layer of the eye wall, the choroid, which is mostly made up of the blood vessels suppling the life source to the outer part of the retina [2]. Recent research has broadened our understanding of the role of the choroid in many retinal and choroidal diseases [3].

Early human eye development is first apparent in the third gestational week, when optic grooves emerge in the cranial neural folds, and is the aftermath of a series of inductive signals. An outgrowth of the forebrain called the optic cup gives rise to the retina from its walls. The walls develop into two layers: the outer thin pigment layer and the thick neural retina. The choroid differentiates from the mesenchyme surrounding the optic cup [4].

Regarding the development of the vasculatures of the choroid and retina, these processes have to be perfectly synchronized to adequately provide the nutritional and oxygen demands of the forming human eye. The choroidal vasculature develops first, and at the seventh week, clearly demonstrates a single layer of capillaries called the choriocapillaris. By the 12th week, intermediate blood vessels begin to form and are linked with the choriocapillaris. The final major vascular system to develop is the retinal vasculature. The retina is predominantly undifferentiated around 5–7 weeks, while the initial vasculature of the primordial choriocapillaris develops by hemo-vasculogenesis. Individual vascular progenitors are present as early as the 7th week, but it is not until 12–14 weeks that these progenitors begin to aggregate and assemble into blood vessels [5]. On the other hand, mouse retinal angiogenesis uses astrocytes as a template, and apparently, the formation of mouse ocular vasculatures is not archetypical of the development of retinal and choroidal vasculatures in humans [6].

Retinoblastoma as the most common intraocular cancer, appearing during childhood, arises from primitive retinal stem cells [7], while uveal melanoma, as the most common cancer in adult eyes, arises from the choroid [8]. If the uveal melanoma has more than 90% of spindle cells or epitheloid cells, it is called spindle or epitheloid cell melanoma, while if having more than 10% of spindle cells and less than 90% of epithelioid cells, it is called mixed cell melanoma [8]. During human retina and choroid development, numerous cells differentiate toward unique cell types using different gene expression patterns and membrane cells signaling in parallel [9,10]. These processes might be again involved in the eye tumorigenesis as a mean to repair the malfunctioned signaling [11]. 

Special proteins called connexins (Cxs) form hemichannels at the level of the cell membrane which connect intracellular and extracellular spaces, or form gap junctions between the neighboring cells [12,13]. As retinal and choroid cells are extensively coupled with gap junctional communications, this might be an important network that enables their metabolic homoeostasis [14,15].

Connexins, as a family of transmembrane proteins, play an important role in cell–cell communication, allowing the direct transfer of small molecules between the cells [16]. Connexin 37 is expressed in endothelial cells and forms intercellular channels that contribute to coordination of the motor tone of the vessels. Mice lacking Cx37 display disturbed early angiogenesis of the retina, thus representing a possible novel target for treatments of retinal diseases [17]. Connexin 40 forms intercellular channels that are important for the electric heart conduction and the vasomotor tone in large vessels. The genetic deletion of Cx40 involves a reduction in vascular growth and capillary density in the neovascularization model of the mouse neonatal retina [18]. Connexin 43 is the most abundant isoform of this family, and in the retina, it is expressed in different cells types (astrocytes, Müller cells, microglia, retinal pigment epithelium, and endothelial cells). Investigations on experimental animal models revealed a role of Connexin 43 in retinal diseases, including macular degeneration, glaucoma, and diabetic retinopathy [19]. Connexin 45 is expressed in bipolar cells and retinal ganglion cells (RGCs) and provides a potential substrate for coordinating network activity. However, its expression does not play a role in gross spatial and temporal propagation properties of retinal waves, but it strongly modulates the firing pattern of individual RGCs, ensuring strongly correlated firing between nearby RGCs and normal patterning of retinogeniculate projections [20].

Contrastingly, Pannexin-1 is a large pore membrane channel with unique conduction properties, ranging from non-selective ion permeability to the extracellular release of signaling molecules [21]. Pannexin1 is expressed along the entire anatomical axis, from optical nerve to retina and cornea in glia, epithelial and endothelial cells, as well as in neurons. In the eye, pannexin 1 is expressed in major divisions including the retina, lens and cornea [22].

However, studies investigating the expression of connexins 37, 40, 43, 45 and pannexin 1 in different tissues of the human eye are rare, especially during development, and most of them are performed on experimental animal models such as mice, rats, or chickens [23,24].

Therefore, we aimed to analyze the expression of connexins 37, 40, 43 and 45 and pannexin 1 in the retina and choroid during human eye development and in the tumor tissue of retinoblastoma and uveal melanoma. We wanted to elucidate Cxs biological importance, since they may represent novel prognostic biomarkers and therapeutic targets for treating retinoblastoma and uveal melanoma, which is an ongoing challenge in the field.

## 2. Results

**Figure 1:**
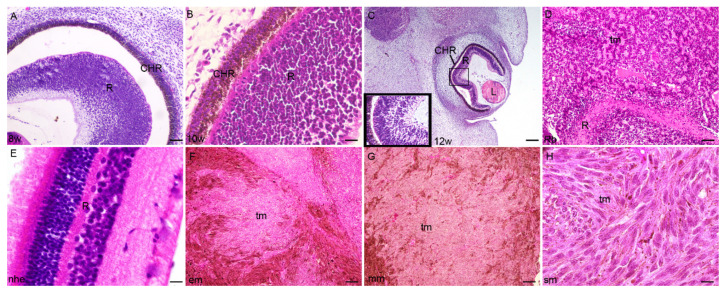
Section through the eye of an 8th-week (8 w) (**A**), 10th-week (10 w) (**B**), and 12th week (12 w) (**C**) human embryo, retinoblastoma (Rb) (**D**), normal human eye (nhe) (**E**), epitheloid melanoma (em) (**F**), mixoid melanoma (mm) (**G**) and spindle melanoma (sm) (**H**); R—retina, CHR—choroidea, tm—tumor tissue, L—lens. Hematoxylin and Eosin staining, Scale bar for (**A**,**D**) = 50 μm, (**B**) = 25 μm, (**E**) = 10 μm, (**C**,**F**–**H**) = 100 μm.

**Figure 2:**
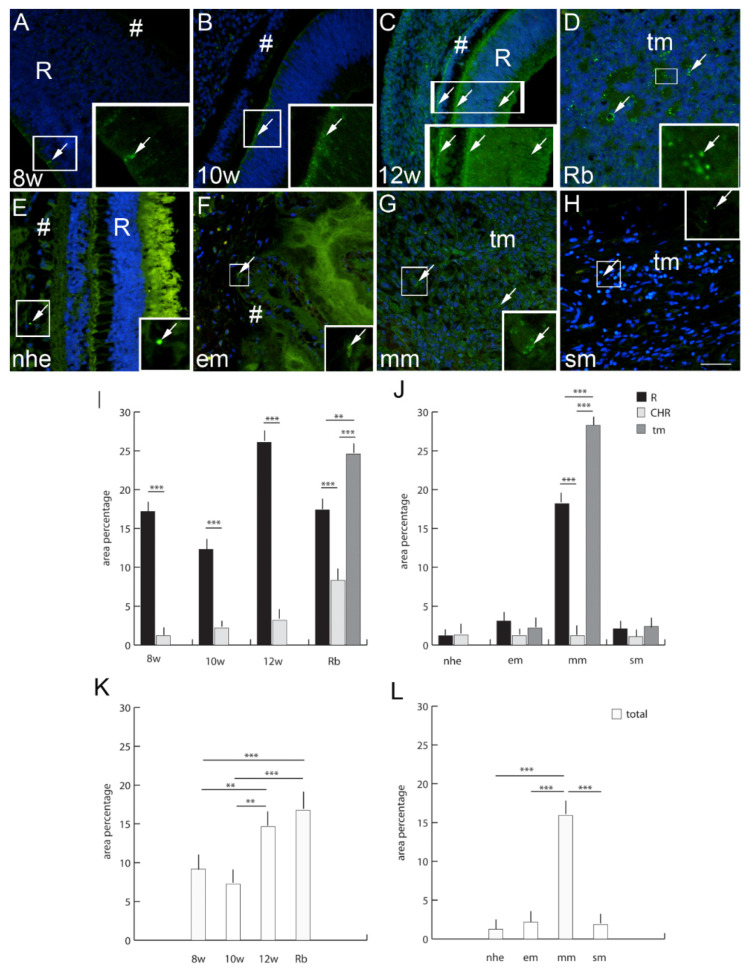
Connexin 37 (Cx37)-positive cells (arrows) can be seen in the eye section of an 8th-week (8 w) (**A**), 10th-week (10 w) (**B**),and 12th week (12 w) (**C**) human embryo, retinoblastoma (Rb) (**D**), normal human eye (nhe) (**E**), epitheloid melanoma (em) (**F**), mixoid melanoma (mm) (**G**) and spindle melanoma (sm) (**H**); R—retina, #—choroidea, tm—tumor tissue. Immunofluorescence staining to Cx37 (green) merged to DAPI (blue nuclei), scale bar 25 μm. The panel with graphs represents the area percentages of Cx37 in the retina (R), choroid (CHR) and tumor (tm) in the 8th-week (8 w), 10th-week (10 w) and 12th-week (12 w) human embryo, retinoblastoma (Rb), normal human eye (nhe), epitheloid melanoma (em), mixoid melanoma (mm) and spindle melanoma (sm) (**I**,**J**), and total area percentage of Cx37 (**K**,**L**). Data are shown as mean ± SD. Significant differences are indicated by ** *p* < 0.01, and *** *p* < 0.001. One-way ANOVA followed by Tukey’s multiple comparisons test (comparison between Rb and uveal melanoma types), *t*-test (comparison between retina and choroid of developmental stages and normal human eye).

**Figure 3:**
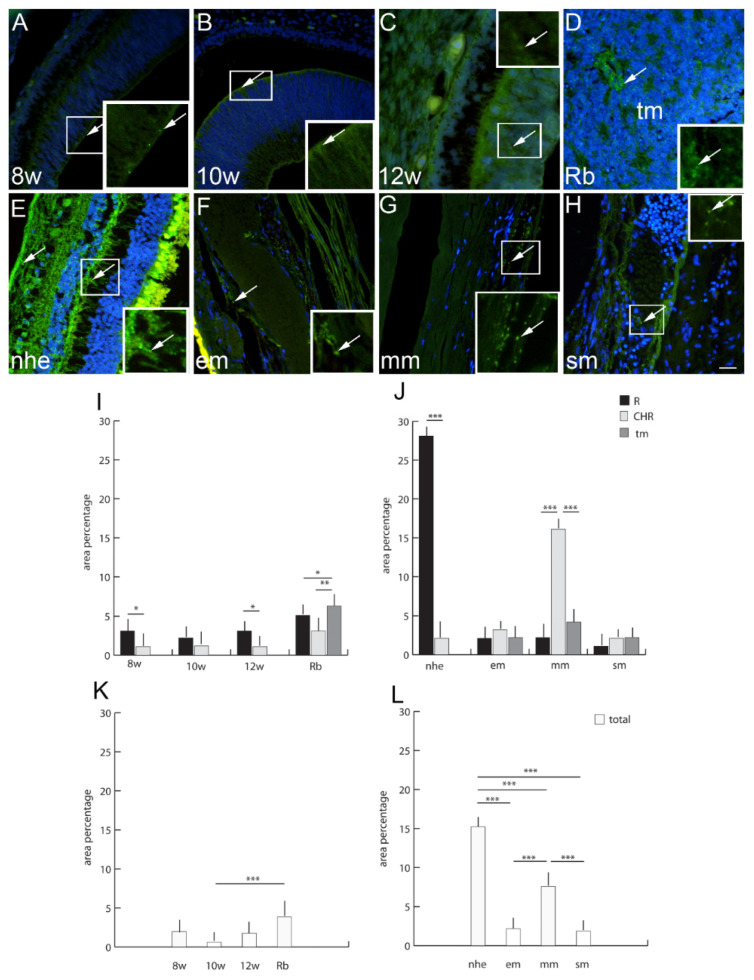
Connexin 40 (Cx40)-positive cells (arrows) can be seen in the eye section of an 8th-week (8 w) (**A**), 10th-week (10w) (**B**) and 12th week (12 w) (**C**) human embryo, retinoblastoma (Rb) (**D**), normal human eye (nhe) (**E**), epitheloid melanoma (em) (**F**), mixoid melanoma (mm) (**G**) and spindle melanoma (sm) (**H**); tm—tumor tissue. Immunofluorescence staining to Cx40 (green) merged to DAPI (blue nuclei), scale bar 25 μm. The panel with graphs represents the area percentages of Cx40 in the retina (R), choroid (CHR) and tumor (tm) in the 8th-week (8 w), 10th-week (10 w) and 12th week (12 w) human embryo, retinoblastoma (Rb), normal human eye (nhe), epitheloid melanoma (em), mixoid melanoma (mm) and spindle melanoma (sm) (**I**,**J**), and total area percentage of Cx40 (**K**,**L**). Data are shown as mean ± SD. Significant differences are indicated by * *p* < 0.05, ** *p* < 0.01, and *** *p* < 0.001. One-way ANOVA followed by Tukey’s multiple comparisons test (comparison between Rb and uveal melanoma types), *t*-test (comparison between retina and choroid of developmental stages and normal human eye).

**Figure 4:**
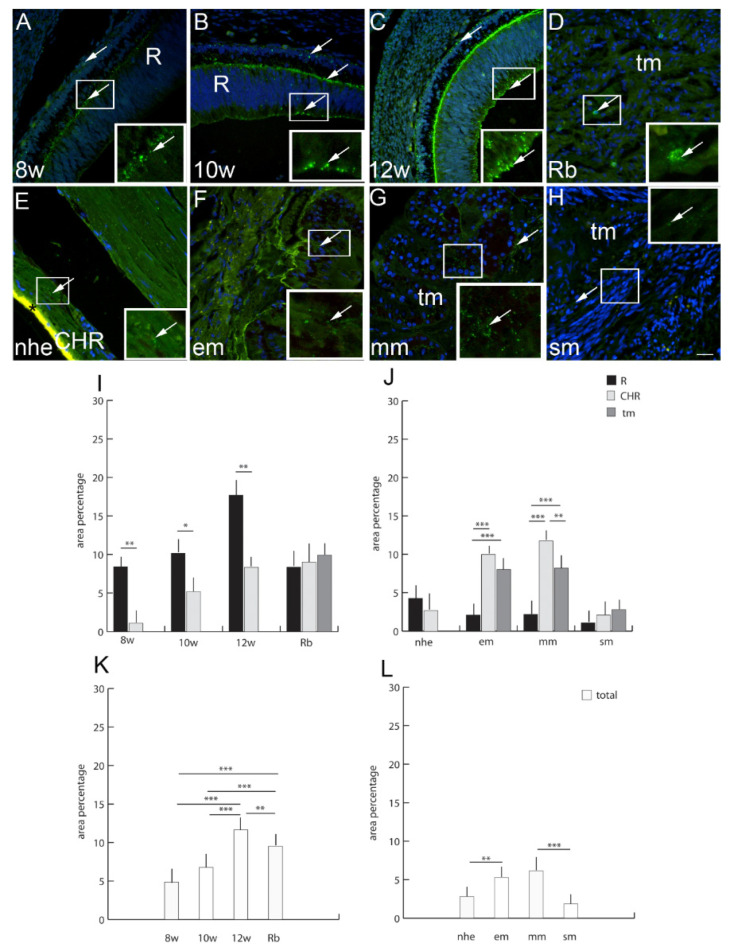
Connexin 43 (Cx43)-positive cells (arrows) can be seen in the eye section of an 8th-week (8 w) (**A**), 10th-week (10 w) (**B**) and 12th week (12 w) (**C**) human embryo, retinoblastoma (Rb) (**D**), normal human eye (nhe) (**E**), epitheloid melanoma (em) (**F**), mixoid melanoma (mm) (**G**) and spindle melanoma (sm) (**H**); R—retina, CHR—choroidea, tm—tumor tissue, *—autofluorescence. Immunofluorescence staining to Cx43 (green) merged to DAPI (blue nuclei), scale bar 25 μm. The panel with graphs represents the area percentages of Cx43 in the retina (R), choroid (CHR) and tumor (tm) in the 8th-week (8 w), 10th-week (10 w) and 12th-week (12 w) human embryo, retinoblastoma (Rb), normal human eye (nhe), epitheloid melanoma (em), mixoid melanoma (mm) and spindle melanoma (sm) (**I**,**J**), and total area percentage of Cx43 (**K**,**L**). Data are shown as mean ± SD. Significant differences are indicated by * *p* < 0.05, ** *p* < 0.01, and *** *p* < 0.001. One-way ANOVA followed by Tukey’s multiple-comparisons test (comparison between Rb and uveal melanoma types), *t*-test (comparison between retina and choroid of developmental stages and normal human eye).

**Figure 5:**
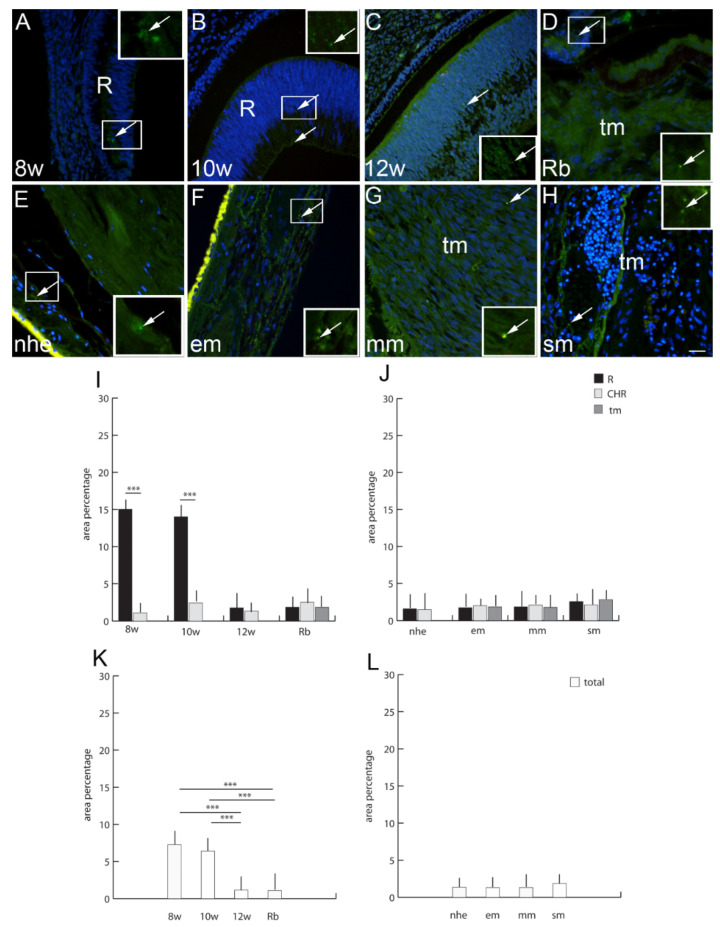
Connexin 45 (Cx45)-positive cells (arrows) can be seen in the eye section of an 8th-week (8 w) (**A**), 10th-week (10 w) (**B**) and 12th-week (12 w) (**C**) human embryo, retinoblastoma (Rb) (**D**), normal human eye (nhe) (**E**), epitheloid melanoma (em) (**F**), mixoid melanoma (mm) (**G**) and spindle melanoma (sm) (**H**); R—retina, tm—tumor tissue. Immunofluorescence staining to Cx45 (green) merged to DAPI (blue nuclei), scale bar 25 μm. The panel with graphs represents the area percentages of Cx45 in the retina (R), choroid (CHR) and tumor (tm) in the 8th-week (8 w), 10th-week (10w) and 12th-week (12w) human embryo, retinoblastoma (Rb), normal human eye (nhe), epitheloid melanoma (em), mixoid melanoma (mm) and spindle melanoma (sm) (**I**,**J**), and total area percentage of Cx45 (**K**,**L**). Data are shown as mean ± SD. Significant differences are indicated by *** *p* < 0.001. One-way ANOVA followed by Tukey’s multiple-comparisons test (comparison between Rb and uveal melanoma types), *t*-test (comparison between retina and choroid of developmental stages and normal human eye).

**Figure 6:**
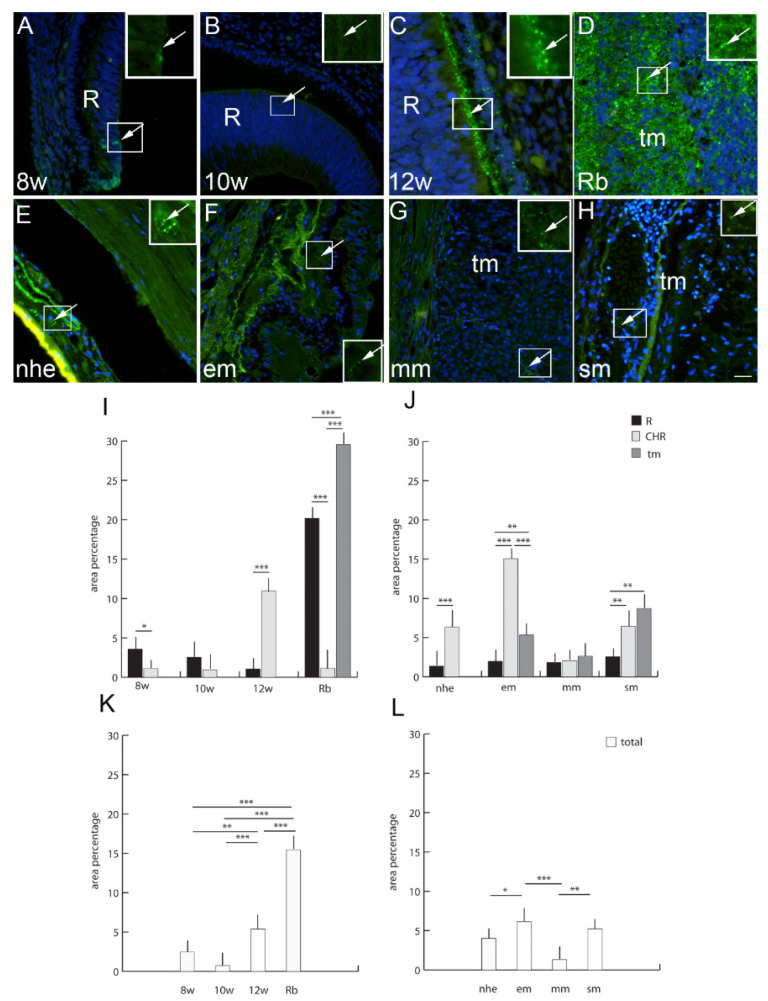
Panexin 1 (Pnx1)-positive cells (arrows) can be seen in the eye section of an 8th-week (8 w) (**A**), 10th-week (10 w) (**B**) and 12th-week (12 w) (**C**) human embryo, retinoblastoma (Rb) (**D**), normal human eye (nhe) (**E**), epitheloid melanoma (em) (**F**), mixoid melanoma (mm) (**G**) and spindle melanoma (sm) (**H**); R—retina, tm—tumor tissue. Immunofluorescence staining to Pnx1 (green) merged to DAPI (blue nuclei), scale bar 25 μm. The panel with graphs represents the area percentages of Pnx1 in the retina (R), choroid (CHR) and tumor (tm) in the 8th-week (8 w), 10th-week (10 w) and 12th week (12 w) human embryo, retinoblastoma (Rb), normal human eye (nhe), epitheloid melanoma (em), mixoid melanoma (mm) and spindle melanoma (sm) (**I**,**J**), and total area percentage of Pnx1 (**K**,**L**). Data are shown as mean ± SD. Significant differences are indicated by * *p* < 0.05, ** *p* < 0.01, and *** *p* < 0.001. One-way ANOVA followed by Tukey’s multiple-comparisons test (comparison between Rb and uveal melanoma types), *t*-test (comparison between retina and choroid of developmental stages and normal human eye).

The expression pattern and quantitative analysis of connexins 37, 40, 43 and 45 and pannexin 1 markers was performed on tissue sections of 8th, 10th and 12th week of development of the human eye, and in retinoblastoma, normal postnatal human eye and uveal eye melanoma, using hematoxylin and eosin (Figure 1) and immunofluorescence staining (Figure 2, Figure 3, Figure 4, Figure 5 and Figure 6).

### 2.1. Developing Human Eye

The inner layer of retina (neural retina) and outer layer of retina (pigmented retina) wall of the gradually disappears by the 8th week of development. In the 8th week of development, the retina is fully surrounded by the highly vascularized and pigmented choroid (Figure 1A). In the 10th week of fetal eye development, the neural retina is most advanced in the posterior part of the optic cup (Figure 1B). In the 12th week of development, the neural retina displayed the first signs of photoreceptive layer differentiation at the retinal outer border, while the ganglion cell layer beneath becomes more apparent (Figure 1C).

### 2.2. Retinoblastoma

Retinoblastoma embryologically initiate from the inner layer of the optic cup with small round blue cells that are more or less differentiated or necrotic with the Homer Wright rosettes with primitive neuroblastic differentiation (Figure 1D).

### 2.3. Epithelioid Melanoma

Epithelioid melanoma cells resemble epithelium because of abundant eosinophilic cytoplasm and enlarged round to oval-shaped nuclei. Epithelioid melanoma cells often lack cohesiveness and demonstrate marked pleomorphism, including the formation of multinucleated tumor cells. Nuclei have a conspicuous nuclear membrane, very coarse chromatin and large nucleoli (Figure 1F).

### 2.4. Myxoid Melanoma

The rare myxoid melanoma manifests large malignant cells amidst a basophilic mucinous matrix. In all cases, the myxoid stroma comprises mesenchymal acidic mucopolysaccharides, as opposed to neutral epithelial mucins. The tumors are essentially amelanotic, although by Fontana Masson preparations, some manifest evidence of melaninogenesis. As there is no cytoplasmic localization of the mucinous material within the tumor cells, it is likely that the myxoid matrix is produced as a response of the stromal cells to the tumor rather than being a product of the tumor cells (Figure 1G).

### 2.5. Spindle Cell Melanoma

Spindle cell melanoma is a rare histological variant of melanoma, characterized by the presence of spindle-shaped melanocytes. On microscopy, it is often mistaken for other skin and soft tissue cancers with spindle cell morphologies (Figure 1H).

### 2.6. Connexin 37

Cx37-positive cells were observed in the human developing eye with a significantly higher expression in the retina in comparison to the choroid (Figure 1). Similarly, to the developing eye, in the retinoblastoma, Cx37 expression was significantly higher in unaffected retina in comparison to choroid. However, Cx37 expression was highest in the retinoblastoma tumor tissue (Figure 1). When comparing normal human eye retina and the choroid, Cx37 expression diminished in comparison to the developing eye and was similar to epitheloid and spindle types of melanoma (unaffected retina, choroid, and tumor tissue) (Figure 1). Only the expression of Cx37 was significantly higher in the unaffected retina and tumor tissue of the mixoid type of melanoma (Figure 1). When we compared the total Cx37 expression regardless of the eye compartment, we could observe that the expression of Cx37 in the retinoblastoma was more similar to that of the 12th week of human eye development and was significantly higher than in the earlier eye development stages. On the other hand, the total Cx37 expression was significantly higher in the mixoid type of melanoma in comparison with the healthy control and epitheloid and spindle type of melanoma (Figure 1).

### 2.7. Connexin 40

Cx40-positive cells were observed in human developing eyes, with a significantly higher expression in the retina at 8th and 12th developmental week in comparison to the choroid (Figure 2). In the retinoblastoma, Cx40 expression was significantly higher in tumor tissue in comparison to unaffected retina and choroid. Overall, Cx40 expression, regardless of the eye compartment, was higher in the retinoblastoma, while the developing eye had a similar expression level. Only in comparison to the 10th week of development was the Cx40 expression significantly lower than in retinoblastoma (Figure 2). When comparing normal postnatal human eye retina and choroid, Cx40 expression significantly increased in comparison to developing eye and overall was significantly higher than in any type of melanoma (Figure 2). The epitheloid and spindle types of melanoma regarding all compartments (unaffected retina, choroidea, and tumor tissue) had similarly low Cx40 expression (Figure 2). Only the expression of Cx40 in the choroid of mixoid type of melanoma was significantly higher in comparison to the unaffected retina and tumor tissue. Additionally, overall Cx40 expression in the mixoid melanoma, regardless of the eye compartment, was significantly higher in comparison to other types of melanoma (Figure 2).

### 2.8. Connexin 43

Cx43-positive cells were observed in the human developing eye with a significantly higher expression in the retina in comparison to the choroidea, especially in the 12th week of development (Figure 3). In the retinoblastoma, Cx43 expression was similar in all eye compartments and without statistically significant differences. However, overall Cx43 expression was significantly higher in the retinoblastoma in comparison to the 8th and 10th developmental week, but significantly lower in comparison to the 12th developmental week (Figure 3). When comparing normal human eye retina and the choroid, Cx43 expression diminished in comparison to developing eye and was similar to the choroid of spindle type of melanoma (Figure 3). Cx43 was significantly higher in the choroid of epitheloid and mixoid type of melanoma in comparison to unaffected retina and tumor tissue (Figure 3). Additionally, the total Cx43 expression, regardless of the eye compartment, was significantly higher in the epitheloid and mixoid type of melanoma in comparison to normal human eye and spindle type of melanoma (Figure 3).

### 2.9. Connexin 45

Cx45-positive cells were observed in human developing eyes with significantly higher expressions in the retina at the 8th and 10th developmental week in comparison to the choroidea (Figure 4). In the retinoblastoma, Cx45 expression was low and similar in all compartments as well as in the 12th week of development (Figure 4). Overall Cx45 expression, regardless of the eye compartment, was higher in the 8th and 10th developmental week in comparison to the 12th week of development and retinoblastoma (Figure 4). When comparing normal human eye retina and choroid to the developing eye, Cx45 expression diminished. However, Cx45 expression in the normal human eye was overall very low and similar to all types of melanoma, both when comparing different compartments and in total rate (Figure 4). 

### 2.10. Panexin 1

Pnx1-positive cells were observed in developing human eyes with the a significantly higher expression in the retina at the 8th developmental week and the choroid in the 12th week of development (Figure 5). In the retinoblastoma, Pnx1 expression was significantly higher in tumor tissue in comparison to the unaffected retina and choroidea. Overall Pnx1 expression, regardless of the eye compartment, was higher in the retinoblastoma compared to all developmental stages (Figure 5). Only in the comparison of the 12th week of development was the Pnx1 expression significantly higher than in earlier developmental stages (Figure 5). When comparing normal human eye retina and the choroid, Pnx1 expression was significantly increased in the choroid and was similar to Pnx1 expression in the spindle type of melanoma. Overall Pnx1 expression in the normal human eye was significantly lower than in epitheloid and spindle types of melanoma (Figure 5). The epitheloid type of melanoma had significantly higher Pnx1 expression, especially in the choroid, while spindle type of melanoma had significantly higher expression in the tumor tissue (Figure 5). Additionally, Pnx1 expression was overall higher in epitheloid and spindle type of melanoma in comparison to the normal human eye and mixoid type of melanoma (Figure 5).

## 3. Discussion

Connexins Cx37, Cx40 and Cx43 are known to be the three mayor vascular connexins in the retina [25]. This is in accordance with our finding of Cx37, Cx40 and Cx43 expression, in the retina and in the vascular eye components, which appeared in a punctate fashion. 

In our study, Cx37 was increasingly expressed in samples of retinoblastoma, especially in the choroid and in the tumor tissue in comparison with the analyzed developing eyes. Increased Cx37 expression in the developing retina of the oldest prenatal eye might be in line with the preparation of the retina for the process of vascularization that occurs in the early postnatal period, which is also in line with our results that suggest a major decrease in expression of Cx37 in adulthood [26]. This could implicate aberrant Cx37 expression in the disruption of the early angiogenesis in humans, which was shown in previous studies conducted on Cx 37^−/−^ mouse models. Additionally, increased Cx37 in the retinoblastoma might be explained by the need of the tumor to develop its own capillary network from the choroid adjacent to the tumor tissue. Cx37 was also highly increased in the mixoid melanoma in comparison to epitheloid, spindle melanoma and normal human eye. This finding might be correlated by increased epithelial to mesenchymal transition (EMT) that is observed in this type of melanoma. Namely, EMT epithelial tumor cells acquire properties of mesenchymal cells, which might contribute to metastatic dissemination and cancer therapy resistance [26]. Increased Cx37 that we observed in our study might be connected to the spreading of the new capillary network in tumor tissue that additionally supports the tumor’s ability for metastasis spreading and a decrease in its sensitivity to therapy. Hamard et al. revealed that, in the venous network of Cx37^−/−^ mice, the loss of Cx37 increased the number of sprouts in the angiogenic area, which contributed to the impaired angiogenic process [27]. It was shown that Cx37 and Cx40 are crucial Cxs in co-regulating angiogenesis [27]. Namely, Cx40 seems to regulate the blood vessel structure and function [28]. However, in our study, we observed that Cx37 and Cx40 had the opposite role in the developmental angiogenesis of the retina. Namely, the expression of Cx37 was much higher than the expression of Cx40 between the 8th and 12th developmental weeks. Our finding is in correlation with the finding of Hamard et al., implying that the decreased expression of Cx40 in embryonal and fetal development might contribute to reduced sprouting of endothelial cells and neovascular formation. Additionally, Cx40 was increased in the retina of the normal human eye as an indicator of vascular network homeostasis. It was also the most abundantly expressed out of all the analyzed markers in the healthy adult sample. However, the higher expression of Cx40 in the choroid of mixoid type of melanoma might be explained by the origin of this type of melanoma from the choroid and its ability to produce a neovascular network for tumor spreading. Namely, mice lacking Cx40 had a decreased number of metastases in the skin melanoma and urogenital carcinoma due to the modulation of the altered growth of vessels in the tumor microenvironment [28]. 

The investigation of differences between expression in the retina and the choroid resulted, as expected from an investigation of the literature, in a higher expression in the retinal tissue during development, but not in the mature eye. Interestingly, there was an upward trend in expression in the samples of developing eyes, which could lead us to believe that Cx43 has an increasingly more important role in intercellular communication due to the rise in the complexity of the developmental process and pathways as time moves forward. Additionally, investigated conexinns were found in the inner and outer surface of the retina and might suggest the involvement in the formation of interneuronal gap junctions. Our results are in line with the study of Sohl et al., who found Cx45 expression in neural retina and Cx43 expression in the ganglion cell layer, while Cx37 expression dominated in the retinal endothelial cells [29]. However, further investigations will need to be conducted to elucidate in which signaling pathways they have roles. 

There is a general increase in Cx43 in the retinoblastoma compared to the 8th and 10th developmental weeks, but a decrease in the retina. This could be the consequence of the retinal progenitor cells giving rise to tumor cells. Since vasculogenesis in the retina begins at the 12th week of development, which would account for the formation of many new gap junctions, the significantly higher expression of this connexin in this period rather than others could mean a novel role in this process [5].

Cx43 was increased in epitheloid and mixoid uveal melanomas, but it was not significantly higher in the retinoblastoma in comparison to the developing eyes. Our results are in line with Ying et al. who also found increased Cx43 expression in the choroidal melanoma [30]. It was also shown that Cx43 might have a role in corneal wound healing, which implies its possible role in repair mechanisms [31]. In our study, we observed an increase in Cx43 in epitheloid and mixoid melanoma, which might be in line with the reparatory function. Namely, cell-adhesion-associated proteins involved in communication with gap junctions seem to be important for tumor cell proliferation, contact inhibition and differentiation. Additionally, Cx43 might have key role in interaction between tumor cells and surrounding immune cells (natural killer, macrophages and dendritic cells) [32]. However, this mechanism is probably out of control in tumor tissue, while the role of the inflammatory process is more involved in the tumor microenvironment and the induction of extravascular metastasis as an alternative pathway for tumor cell spreading. Namely, based on the role of Cx43 mimetic peptides in reducing inflammation through blocking proinflammatory mediator production and inhibition of increased ATP release [14,33], we can speculate that increased Cx43 expression in our study might contribute to inflammatory response. Therefore, increased Cx43 expression could be regarded as a prognostic biomarker and possible therapeutical target in epitheloid melanoma. This is in line with the knowledge that epitheloid melanoma have poor prognosis. 

The expression of Cx45 was the highest in the early eye development, but it diminished from the 12th week of development and in the postnatal period. We did not find increased Cx45 expression neither in the retinoblastoma nor in the uveal melanoma samples, which could lead us to conclude that it does not take part in these pathological processes. In contrast, the study of Guo et al. revealed high expression of Cx45 in the adult Sprague Dawley rats’ retina in control animals, and decreased Cx45 expression after light damage retina injury [34]. However, we did not find any studies exploring Cx45 in the retinoblastoma and uveal melanoma. In our previous study, we also find Cx45 not to be relevant either in the yotari mice inner ear development (E13.5 and E15.5) or in the 8th week of developing human inner ear [12]. Our result might imply that Cx45 is not as relevant in the fetal and postnatal period as it is during early human eye development (8th and 10th developmental week) when normal patterning is ensured, especially in the retina.

Pnx1 regulates cellular processes in melanoma cells, including proliferation, migration, and invasion/metastasis during melanoma tumorigenesis [35]. Pnx1 is highly expressed in the retinal and tumor tissue of retinoblastomas in comparison to the developing human eye. Of all types of melanoma, Pnx1 is highly expressed in the choroid of the epithelioid type of melanoma. This finding might indicate a role for Pnx1 in the regulation of the melanoma cell metabolic profile. Namely, Pnx1 seems to correlate with β-catenin; thus, blocking Pnx1 in melanoma decreases its level and causes the suppression of β-catenin transcriptional activity. This could lead to reduced proliferation, migration and invasion/metastasis during melanoma tumorigenesis. Although some studies have shown how blocking Cx43 is beneficial for tumor spreading, other studies have also indicated that blocking Pnx1 might also beneficially contribute to better cancer therapy [36]. This suggests that possible Pnx1 novel therapeutical targeting might improve the treatment of uveal melanoma.

The limitation of our study is the use of human embryonic and fetal samples, and therefore, it is difficult to draw a conclusion about the underlying mechanism with only a descriptive study. Animal models would certainly significantly improve our understanding of mechanisms and the role of investigated gap junctions. Additionally, an investigation of specific connexin expression in different cell types in the retina and the choroid will be necessary to establish their functional role.

In conclusion, our study has shown the involvement of Cx37 and Cx40 expression in normal and pathological vascularization, and Cx43 expression in the inflammatory response. Whereas Cx45 is involved in early stages of eye development, Pnx1 might influence cell metabolism. We also observed significant differences in the expression of analyzed Cxs and Pnx1 between the different types of tumors, which can be used in their fine distinction. Although the functional details in studies of preserved postmortem human tissue are difficult to clarify, it is required to compare the gene expression seen in rare samples of human tissue, as presented in our research, to that of mice, since they are not representative. Therefore, Cxs and Panx1 could serve as potential molecular biomarkers of tumor prognosis and possibly as targets for future novel therapeutical approaches.

## 4. Materials and Methods 

### 4.1. Tissue Processing and Procurement

The embryonic tissues were obtained from the archive of Department of Anatomy, Histology and Embryology, University of Split School of Medicine. We used 15 conceptuses at the 8th, 10th and 12th week of development (five for each stage) according to the Carnegie staging system based on morphology, external measurements (crown-rump length) and menstrual data as we previously described [37,38] in accordance with the Helsinki Declaration and its updates. Prior to the application of immunofluorescence, proper tissue preservation was confirmed by H&E staining of every 10th section.

The samples of normal human eye (five samples), retinoblastoma (five samples) and melanoma (fifteen samples) were obtained at the Department of Pathology of University Hospital of Split. The study was carried out with the approval of the Ethical Committee of the General Hospital Zadar (ur.br. 02-1237/22-15/22) in accordance with the Helsinki Declaration and its updates. All acquired samples were evaluated by two pathologists who classified the diagnoses. Only well-preserved tissues were used after exterior examination and any macerated or poorly maintained material was discarded. Standard hematoxylin and eosin staining was performed with each tissue block to confirm appropriate material preservation.

### 4.2. Tissue Preparation for Indirect Immunofluorescence

The tissue samples were fixed in 4% paraformaldehyde dissolved in phosphate-buffered saline (PBS), dehydrated in graded ethanol, and paraffin-embedded. Serial 5µm-thick sections were cut in a transversal plane and mounted on glass slides. The deparaffinization of tissue in xylol and rehydration in graded ethanol and distilled water were necessary before being heated in a citrate buffer for 20 min at 95 °C in a water steamer and gradually cooled to room temperature. Next, protein blocking buffer (ab64226, Abcam, Cambridge, UK) was applied for 20 min to prevent non-specific staining. All samples were then incubated with suitable primary antibodies (Table 1) overnight in a humidity chamber. The following day, they were incubated with fluorescent-dye-labeled secondary antibodies (Table 1) for two hours. Lastly, the nuclei were stained using 4’,6-diamidino-2-phenylindole (DAPI) and then cover-slipped (Immuno-Mount, Thermo Shandon, Pittsburgh, PA, USA). No immunoreactivity was observed when primary antibodies were omitted from the protocol.

Images of developing, normal and eyes with retinoblastoma and different types of uveal melanoma used for analysis were taken at 40× magnification with a fluorescence microscope (Olympus BX51, Tokyo, Japan) equipped with a Nikon DS-Ri1 camera (Nikon Corporation, Tokyo, Japan). For the quantitative analysis of connexin and pannexin immunoexpression, ten non-overlapping representative visual fields of identical exposure time were captured. Granular and/or diffuse cytoplasmatic staining was interpreted as positive Cx40, Cx43, Cx37, Cx45 and Panx1 immunoexpression. ImageJ software (National Institutes of Health, Bethesda, MD, USA) was utilized for the quantitative evaluation of immunoreactivity. Before analysis, image preparation was performed using subtraction of the median filter and color thresholding to measure the area covered by positive signal. Green staining was interpreted as positivity for Cx37, Cx40, Cx43, Cx45, and Panx1 immunoexpression. GraphPad Software (GraphPad Software, La Jolla, CA, USA) was utilized for statistical analyses, with the probability level of *p* < 0.05 being regarded as statistically significant. A one-way ANOVA test followed by post hoc Tukey’s test was used to compare immunoexpression in order to determine significant differences among groups. The data are summarized as mean ± SD. Three investigators analyzed the images independently, while three to four tissue samples were used per group in each replicated experiment (n ≥ 3).

## Figures and Tables

**Table 1:** Antibodies used for immunofluorescence.

Antibodies		Host	Dilution	Source
Primary	Anti-Cx37/GJA4 ab181701	Rabbit	1:500	Abcam (Cambridge, UK)
	Anti-Cx40/GJA5 ab213688	Rabbit	1:100	Abcam (Cambridge, UK)
	Anti-Cx43&GJA1 ab87645	Goat	1:200	Abcam (Cambridge, UK)
	Anti-Cx45/GJA7 ab135474 Anti-pannexin 1/PANX1	Rabbit Rabbit	1:100 1:300	Abcam (Cambridge, UK) Merck KGaA (Darmstadt, Germany)
Secondary	Anti-Goat IgG, Alexa Fluor^®^ 488, ab150129	Donkey	1:400	Abcam (Cambridge, UK)
	Anti-Rabbit IgG, Alexa Fluor^®^ 488, 711-545-152	Donkey	1:400	Jackson Immuno Research Laboratories, Inc. (Baltimore, PA, USA)

Data Acquisition, Semi-Quantitative and Statistical Analysis.

## Data Availability

Not applicable.
